# Evaluation of Gynecological Mass Lesions Using Magnetic Resonance Imaging: An Observational Study

**DOI:** 10.7759/cureus.104200

**Published:** 2026-02-24

**Authors:** Tanushri Ghosh, Subhankar Choudhury, Anand Kumar

**Affiliations:** 1 Department of Radiodiagnosis, Hi-Tech Medical College and Hospital, Rourkela, IND; 2 Department of Pharmacology, Hi-Tech Medical College and Hospital, Rourkela, IND; 3 Department of Forensic Medicine and Toxicology, Hi-Tech Medical College and Hospital, Rourkela, IND

**Keywords:** gynecological masses, magnetic resonance imaging, non-neoplastic masses, ovarian neoplasms, uterine neoplasms

## Abstract

Background: Gynecological masses in females comprise benign and malignant masses. These lesions have become a great concern for gynecologists and radiologists because the pelvic organs are aligned in such proximity that any masses arising from these organs may share similar signs and symptoms. Though ultrasonography is the first preference to investigate these masses, it has limited value because of its poor acoustic windows, poor depth of penetration, and operator dependency. Computed tomography is devoid of soft tissue contrast and becomes more complex when discriminating pelvic lesions from decompressed loops of intestine. However, MRI has been found to be more useful than other imaging modalities because it provides better contrast resolution, which facilitates precise tissue characterization and enhanced anatomical depiction; therefore, it is more specific and accurate than ultrasonography and computed tomography for diagnosing gynecological masses.

Materials and methods: This retrospective study was conducted in the Department of Radiodiagnosis at Hi-Tech Medical College and Hospital, Rourkela, Odisha, after obtaining approval from the Institutional Ethics Committee. After optimizing the minimal sample size, a total of 106 cases were selected for this study during the period of February 2024 to July 2025. Patients who underwent MR imaging of the pelvis on the advice of the referring gynecologist after suspicion of a gynecological mass were included in this study.

Results: Out of 106 cases, 69 (65.09%) were neoplasms, and 37 (34.91%) were non-neoplastic lesions. Among the neoplasms, 52 (49.06%) were benign masses and 17 (16.04%) were malignant masses. Among benign masses, fibroids were identified in 31 cases (29.24%), and benign ovarian tumors in 21 cases (19.81%). Of the malignant lesions, nine (8.49%) were cervical carcinomas, seven (6.6%) were endometrial carcinomas, and one was an ovarian epithelial carcinoma. Among the non-neoplastic lesions, the incidence of endometriosis at 19 (17.92%) was the highest. It was followed by seven cases of adenomyosis (6.6%). Other non-neoplastic masses were ovarian cysts, endometrial polyps, hematocolpos, and retained products of conception.

Conclusion: MRI is the sole imaging technique that effectively assesses gynecological masses and addresses radiological challenges. Its non-invasive nature renders it preferable for patients, particularly when ethical concerns arise regarding histopathological evaluations. Additionally, MRI aids in the characterization, classification, and future management of gynecological masses.

## Introduction

In contemporary medical practice, magnetic resonance imaging (MRI) is the principal modality for assessing gynecological lesions in females. Gynecological masses in females are either neoplastic or non-neoplastic. Neoplastic lesions can be further categorized as benign or malignant. These masses can originate from various pelvic structures, such as the uterus, ovaries, fallopian tubes, and soft tissues [1]. Benign masses predominantly consist of fibroids, as well as ovarian epithelial, stromal, and germ cell tumors. The documented malignancies, in descending order of incidence, are cervical, ovarian, endometrial, vulval, and vaginal cancers. Endometriosis, ovarian cysts, adenomyosis, polyps, imperforate hymen, and other adnexal inflammatory diseases are classified as non-neoplastic masses in various studies [2].

Currently, ultrasonography is often the preferred first-line imaging technique for gynecological lesions because it is non-radiative and cost-effective; however, the proximity of these masses to multiple pelvic and abdominal structures presents diagnostic challenges due to overlapping imaging characteristics [3]. Ultrasonography is also limited by operator skill, patient movement, and low signal-to-noise ratio, which hinder its ability to accurately characterize malignancies due to inadequate acoustic windows and penetration depth; consequently, it may fail to detect invasion of surrounding structures and lymph node enlargement in cancerous lesions [4].

Computed tomography also presents several drawbacks, including the utilization of ionizing radiation that poses risks to women of reproductive age, insufficient soft tissue contrast complicating the differentiation between benign and malignant lesions, and challenges in identifying the source of pelvic masses, as well as the potential interference of decompressed intestines in diagnosing adnexal pathology [5].

By achieving remarkably high contrast resolution for soft tissues, MRI enhances accurate identification of normal pelvic anatomy and diverse pathological conditions. Its non-reliance on ionizing radiation renders it safe across nearly all demographic categories. MRI is capable of yielding high-resolution images in various planes without necessitating patient movement, thereby providing a comprehensive assessment of gynecological masses and their anatomical relationships with adjacent structures, which is instrumental in the precise staging and therapeutic planning for these masses [6]. Moreover, MRI proficiently reveals the components found within lesions, including adipose tissue, hematoma, mucin, protein, and several other materials. Ultimately, MRI can also discern between residual and recurrent disease during follow-up assessments and can detect remission [6]. Although histopathology remains the primary method for definitive diagnosis of these masses, ethical concerns arise regarding patient perspectives, particularly when considering cultural attitudes and the invasive nature of the procedure [7]. Limitations may also exist within laboratory capabilities, encompassing logistical challenges and the availability of skilled pathologists. In such scenarios, MRI may indeed serve as the most reliable investigative modality.

The purpose of this study was to explore the precision of MRI for diagnosing gynecological masses in the absence of histopathological examination. The sole aim of our study was to establish the role of MRI in diagnosing gynecological masses in terms of their morphology, location, and relationship with surrounding structures. Our objectives were to identify and describe the MRI features of benign, malignant, and non-neoplastic gynecological masses. By exploring recent research articles and clinical observations, the findings of this study may summarize the characteristics of gynecological masses to aid in their future management.

## Materials and methods

The current retrospective observational analysis was carried out within the Department of Radiodiagnosis, Hi-Tech Medical College and Hospital, Rourkela, Odisha. Prior to submitting the relevant documents to us, the medical records department made sure that the patients’ names and identification numbers were erased from all materials related to this research. The investigation commenced on September 15, 2025, and concluded on October 18, 2025.

MRI examinations were performed on patients in the supine position using a 1.5 T scanner (Signa; Milwaukee, WI: General Electric Medical Systems, Inc.) after a 6-h fast, which mitigated artifacts and enhanced image quality. The MRI protocol incorporated critical sequences, including T1-weighted (T1W) and T2-weighted (T2W) imaging and short tau inversion recovery, in axial, sagittal, and coronal orientations, thereby facilitating precise anatomical and pathological evaluation essential for diagnosing gynecological masses. Through T1W and T2W imaging, notable distinctions among pelvic organs and lesions were established, enabling comprehensive assessment of the shapes, sizes, margins, and anatomical context of the masses.

Inclusion and exclusion criteria

Female patients aged 14 to 75 years who sought outpatient care and underwent magnetic resonance imaging (MRI) of the pelvic region at the Department of Radiodiagnosis, Hi-Tech Medical College and Hospital, Rourkela, upon the recommendation of a referring gynecologist from the same institution, following clinical suspicion of a pelvic mass based on clinical examination and ultrasonography, between February 1, 2024, and July 31, 2025, were included in this investigation. Individuals aged below 14 years and above 75 years, as well as inpatients and those who had undergone surgical procedures, were excluded from the study.

Sample size

Although an initial approximate sample size was estimated using Cochran’s formula based on previously reported incidence rates, the study ultimately employed consecutive sampling, and all eligible patients presenting during the study period from February 1, 2024, to July 31, 2025, were included subsequent to the implementation of the defined inclusion and exclusion parameters [8-10]. A total of 119 female subjects underwent MRI of the pelvis following the identification of a potential gynecological mass by the referring gynecologists. Out of these 119 instances, 13 were removed from the analysis because of insufficient data. Consequently, we included all remaining cases in our research, resulting in a final sample size of 106.

Data collection and analysis

Upon meticulous examination of the medical documentation, a comprehensive dataset was assembled, encompassing parameters such as patient demographics, the prevalence of gynecological masses across distinct age cohorts, the dimensions and characteristics of the masses, the predominant clinical manifestations associated with these masses, and the individual occurrences within each mass category. Data collection was conducted using a meticulously designed pro forma.

The gathered data were subsequently organized into a Microsoft Excel worksheet 2021 (Redmond, WA: Microsoft Corp.) for analytical purposes. Descriptive statistical methods were employed using numerical values and percentages to assess categorical variables. Subsequently, the requisite tables and figures were produced through the Microsoft Excel worksheet. The relevant MRI images were procured from the archives of the computer system within the department of radiodiagnosis, ensuring the anonymity of patients was preserved by the medical personnel involved.

## Results

In the current study, patients’ demographic characteristics, including age group, marital status, religion, employment status, and socioeconomic status, were initially evaluated. A total of 56 females (52.83%) presenting with gynecological masses were in their fourth and fifth decades of life. Among them, 64 (60.38%) were married at the time of their diagnosis. Furthermore, 61 (57.55%) identified as adherents of the Hindu faith. Additionally, 74 (69.81%) were either unemployed or engaged in homemaking, whereas only 21 (19.81%) were classified in the higher socioeconomic tier according to the Updated B.G. Prasad Scale 2025 (Table 1) [11].

**Table 1:** Demographic details of patients.

Characteristics	Categories	Frequency	%
Age group (years)	14-20	3	2.83
	21-30	13	12.26
	31-40	25	23.58
	41-50	31	29.25
	51-60	21	19.81
	61-70	9	8.49
	71-80	4	3.78
	Total	106	-
Marital status	Single	15	14.15
	Married	64	60.38
	Separated	7	6.60
	Divorced	11	10.38
	Widow	9	8.49
	Total	106	-
Religion	Hindu	61	57.55
	Muslim	28	26.41
	Christian	9	8.49
	Sikh	5	4.72
	Jain	3	2.83
	Total	106	-
Employment status	Not employed (housewife)	74	69.81
	Part-time employment	23	21.70
	Full-time employment	9	8.49
	Total	106	-
Socioeconomic status	Class I (upper)	11	10.38
	Class II (upper middle)	10	9.43
	Class III (middle)	24	22.64
	Class IV (lower middle)	29	27.36
	Class V (lower)	32	30.19
	Total	106	-

**Figure 1:**
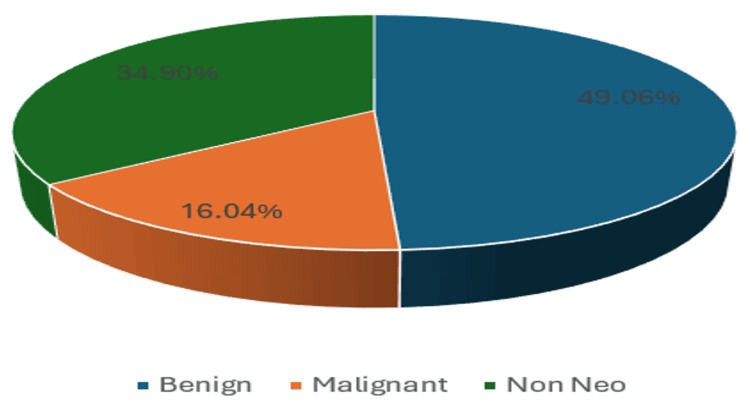
Distribution of gynecological masses. Non Neo: non-neoplastic

At the time the diagnosis was formulated, various clinical and laboratory parameters were correlated with the MRI findings, which led to the final diagnosis. These parameters included age, medical history, clinical manifestations, and standard biochemical assays. In our study, from a total of 106 cases, 69 lesions (65.09%) were classified as neoplastic, whereas 37 (34.90%) were deemed non-neoplastic. Among the 69 neoplastic cases, 52 cases (49.06% of the total) were identified as benign, whereas 17 cases (16.04% of the total) were classified as malignant (Figure 1).

Out of a total of 52 cases of benign neoplasm, 39 were identified within the reproductive age cohort, whereas the remaining cases were located within the postmenopausal demographic. Among the 17 malignant lesions encountered, a mere four cases were detected within the reproductive age group. Conversely, in the context of non-neoplastic cases, the highest incidence was observed in individuals aged 30-45 years (Table 2).

**Table 2:** Distribution of gynecological masses according to age group.

Age group (years)	Benign mass	Malignant mass	Non-neoplastic mass	Total cases
14-18	0	0	3	3
19-30	6	0	7	13
31-40	16	0	9	25
41-45	17	4	10	31
46-60	8	9	4	21
61-70	3	3	3	9
>70	2	1	1	4
Total cases	52	17	37	106

In the aggregate of 106 clinical encounters, the predominant presenting complaint was abdominal pain (29 instances), followed by menstrual irregularities (23 instances), abdominal distention (16 instances), postmenopausal hemorrhage (13 instances), vaginal discharge (10 instances), and infertility (eight instances). Seven cases were identified incidentally, all of which were classified as non-malignant masses. Among the 52 benign neoplasms, the most frequently observed clinical presentations included abdominal pain (17 instances) and menstrual irregularities (14 instances). Similar patterns were observed among the non-neoplastic masses. However, in the context of malignant masses, the most prevalent clinical presentations were postmenopausal hemorrhage and vaginal discharge (Table 3).

**Table 3:** Most common clinical presentations of gynecological masses. p/v: per vaginal

Most common clinical symptom	Benign neoplasms	Malignant neoplasms	Non-neoplastic masses	Total number of cases
Abdominal pain	17	2	10	29
Menstrual irregularities	14	2	7	23
Abdominal distention	12	2	5	19
Postmenopausal bleeding	2	6	2	10
Discharge p/v	0	5	5	10
Infertility	3	0	5	8
Diagnosed in routine checkup	4	0	3	7
Total	52	17	37	106

**Figure 2:**
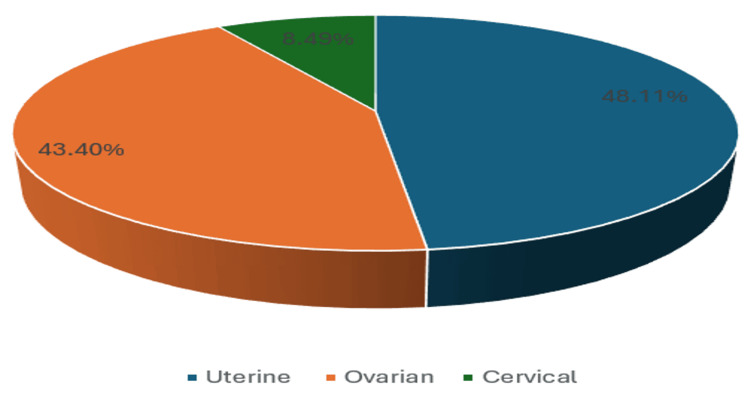
Distribution of masses according to organ of origin.

**Figure 3:**
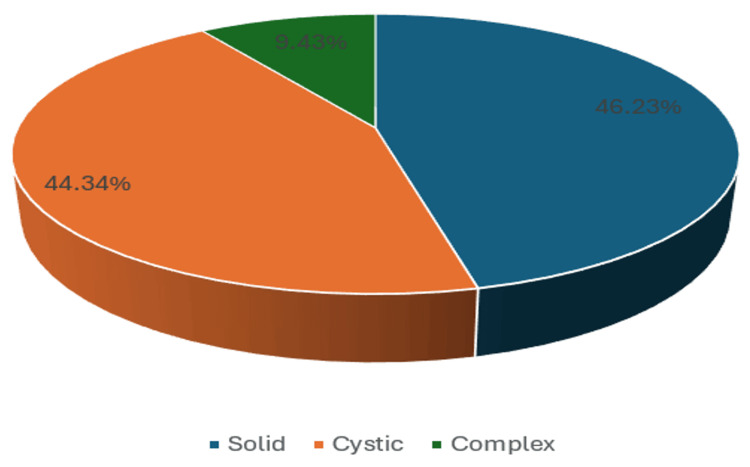
Comparison of pelvic masses according to internal consistency.

Among the total 106 cases, 51 cases (48.11%) had uterine masses, 46 cases (43.40%) had ovarian masses, and nine cases (8.49%) had cervical masses (Figure 2). In the analysis of pelvic masses categorized by internal consistency, a total of 49 cases (46.23%) were identified as solid masses, 47 cases (44.34%) were classified as cystic masses, and 10 cases (9.43%) were recognized as complex masses (Figure 3).

When the sizes of the masses were subjected to comparison, approximately 41.51% of the specimens were observed to fall within the dimension range of 5-10 cm. Most of these specimens were identified as either benign epithelial ovarian tumors, fibroids, endometriosis, or endometrial carcinomas. This group was succeeded by masses that were measured between 3-5 cm (32.08%). Predominantly, these masses consisted of fibroids, ovarian tumors, cervical carcinomas, and adenomyosis. A subset of cases that involved ovarian epithelial tumors, endometriomas, and hematometra associated with imperforate hymens exhibited dimensions exceeding 10 cm in length. Endometrial polyps, certain ovarian cysts, and select fibroids were noted to be less than 3 cm (Table 4).

**Table 4:** Distribution of gynecological mass according to size.

Gynecological mass (cm)	Benign mass	Malignant mass	Non-neoplastic mass	Total, n (%)
<3	4	0	7	11 (10.38)
3-5	14	11	9	34 (32.07)
5-10	23	6	15	44 (41.51)
>10	11	0	6	17 (16.04)
Total	52	17	37	106

**Figure 4:**
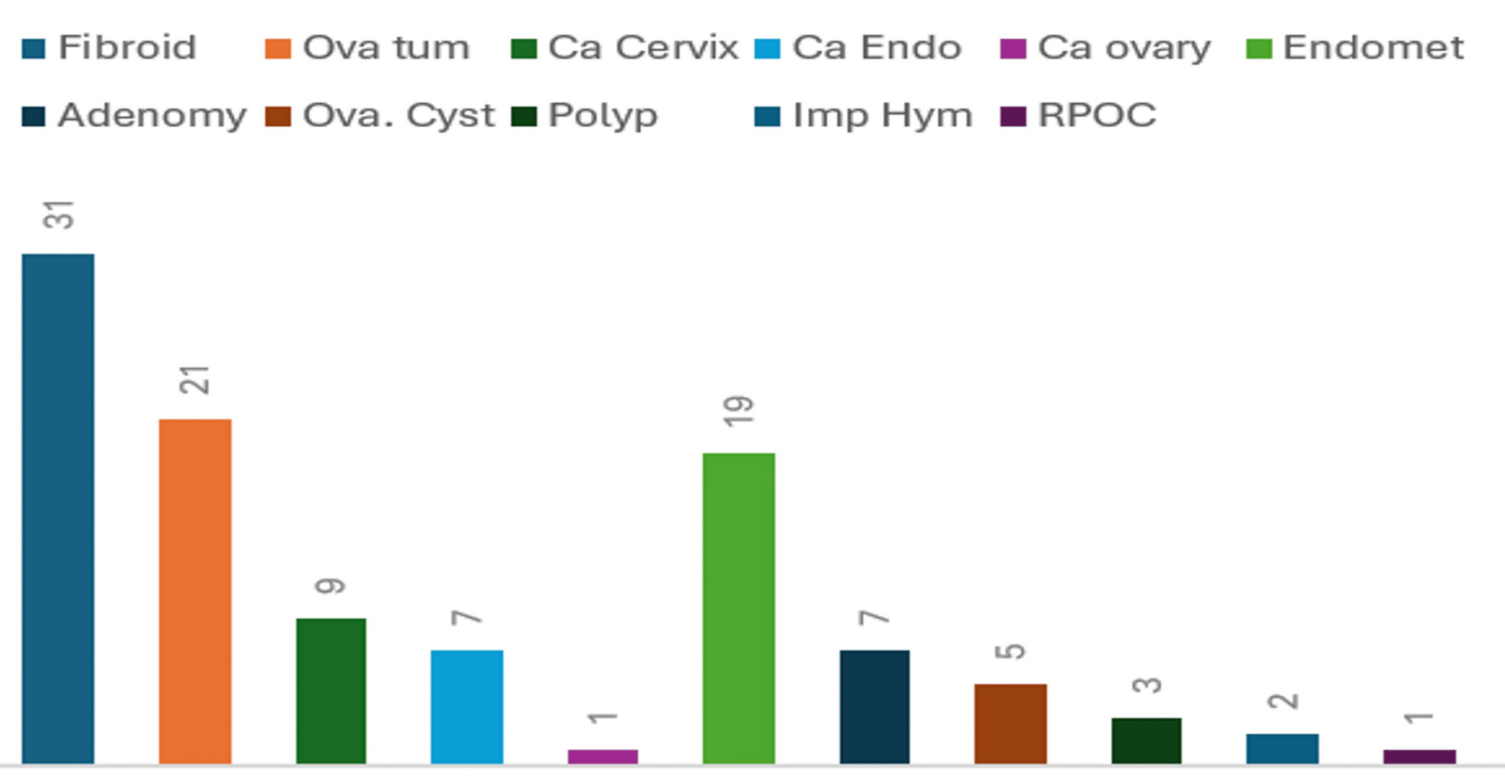
Distribution of gynecological masses according to their individual incidences.

Of the 52 benign neoplasms identified, 31 were classified as uterine fibroids (comprising 20 intramural and 11 sub-mucosal variants), whereas 21 were categorized as benign ovarian neoplasms (including 11 serous cystadenomas, nine mucinous cystadenomas, and one ovarian fibroma). Of the malignant neoplasms, diagnoses included nine instances of cervical carcinoma, seven occurrences of endometrial carcinoma, and one case of mucinous cystadenocarcinoma. Within the 37 non-neoplastic masses, 19 were attributed to endometriosis, seven were attributed to adenomyosis, five were identified as ovarian cysts, three were cases of hematometra associated with an imperforate hymen, two were classified as endometrial polyps, and one case was indicative of the retained products of conception (RPOC) (Figure 4). Among the malignant neoplasms, when International Federation of Gynaecology and Obstetrics (FIGO) staging was interpreted according to MRI findings, all masses were in or above stage II at the time of imaging (Table 5).

**Table 5:** Correlation of MRI findings to FIGO staging in malignant neoplasms. FIGO: International Federation of Gynaecology and Obstetrics

S. no.	Case no.	Diagnosis	MRI staging
1	9	Cervical carcinoma	IIB
2	14	Cervical carcinoma	IVA
3	23	Endometrial carcinoma	IIA
4	26	Cervical carcinoma	IIB
5	32	Cervical carcinoma	IIIA
6	34	Endometrial carcinoma	IIIB
7	41	Endometrial carcinoma	IVA
8	52	Cervical carcinoma	IIA
9	56	Endometrial carcinoma	IIIA
10	67	Endometrial carcinoma	IIA
11	69	Cervical carcinoma	IIIB
12	73	Cervical carcinoma	IIA
13	80	Cervical carcinoma	IVA
14	84	Endometrial carcinoma	IIIA
15	89	Mucinous cystadenocarcinoma	IIIC
16	92	Endometrial carcinoma	IVA
17	97	Cervical carcinoma	IIIA

MRI observations of various gynecological masses

**Figure 5:**
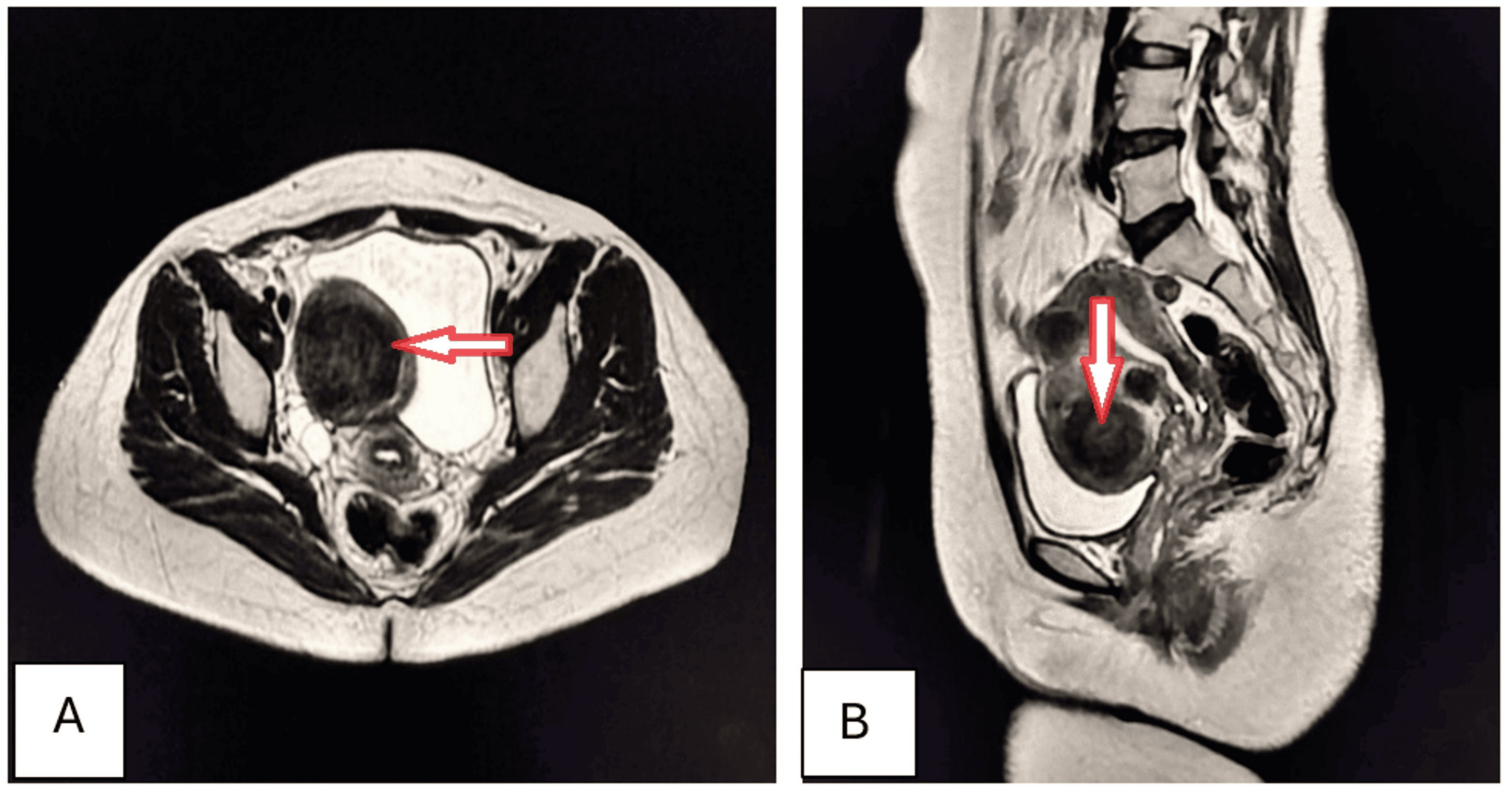
Intramural uterine fibroid (arrows). Axial (A) and sagittal (B) T2W MRI imaging shows a well-defined, round-to-oval, homogenously hypointense mass in the anterior myometrium, consistent with intramural fibroids. It is surrounded by similar, smaller fibroids. Mass effect is noted as indentation over the superior aspect of the bladder and into the endometrial canal. T2W: T2-weighted

A 32-year-old female presented to the outpatient department (OPD) with complaints of episodic, dull, boring pain and intermittent menorrhagia for seven months. These were associated with increased frequency of urination, constipation, and painful intercourse. Laboratory investigations revealed the presence of normocytic normochromic anemia. Ultrasonography revealed a well-defined hypoechoic mass in the myometrial tissue. Axial and sagittal T2-weighted (T2W) MRI confirmed the presence of intramural fibroid (Figures 5A, 5B).

**Figure 6:**
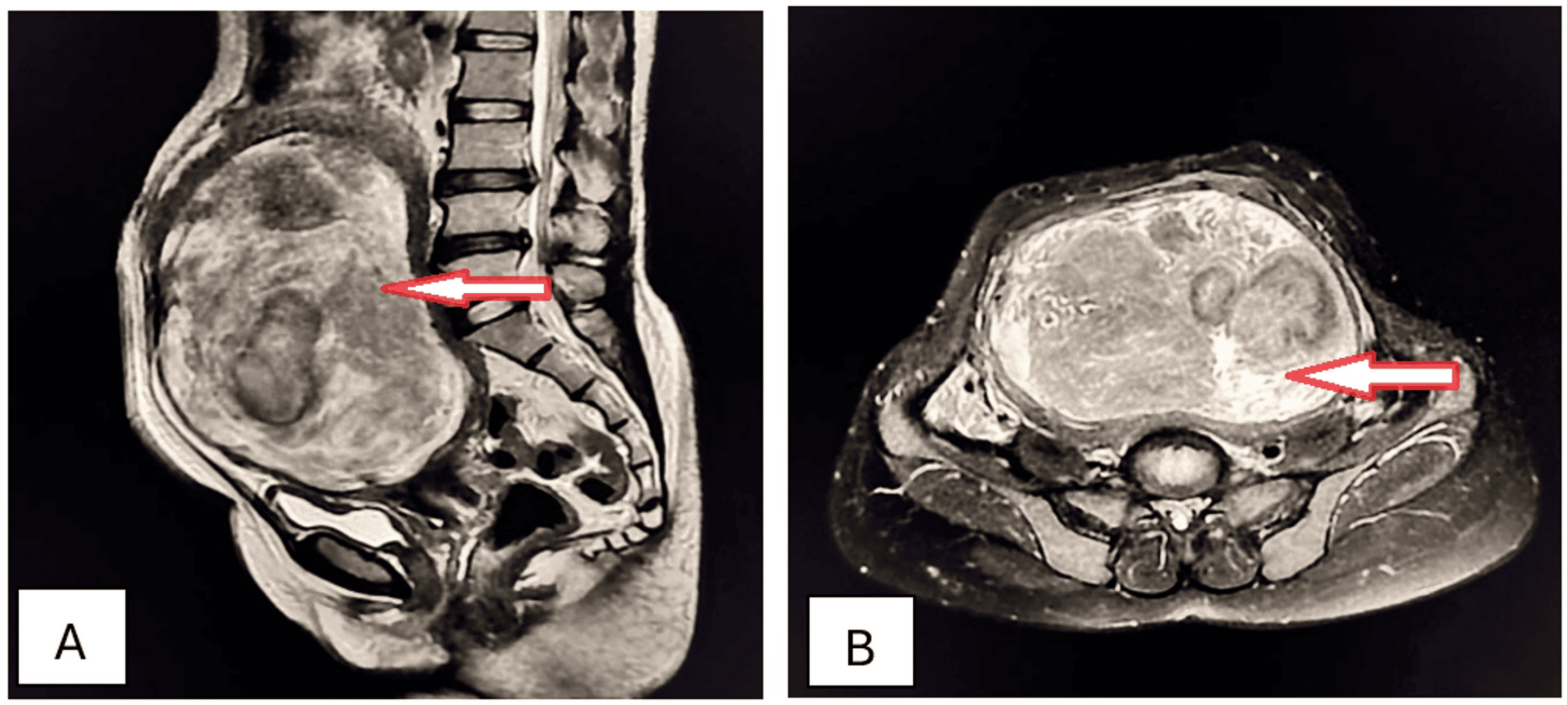
Intramural uterine fibroid with hyaline degeneration (arrows). Sagittal (A) and axial (B) T2W MRI imaging shows a huge, well-defined, inhomogeneous, encapsulated, round-to-oval, hypointense, solid abdominopelvic mass within the myometrium, causing myometrial thinning and exhibiting mixed signal intensity consistent with an intramural fibroid with hyaline degeneration. T2W: T2-weighted

A 41-year-old female visited the outpatient department with complaints of severe menorrhagia, chronic severe pelvic pain, backache, and lower abdominal fullness for the preceding 15 months. These were associated with an increased frequency of micturition and chronic constipation. Physical examination disclosed a large, firm, irregular, palpable mass in the lower abdomen. A complete blood count revealed microcytic hypochromic anemia. Ultrasonography showed the presence of a well-defined, solid, hypoechoic, heterogeneous mass arising from the myometrium, causing significant uterine enlargement and contour distortion. Sagittal and axial T2W MRI imaging identified the presence of intramural fibroid with hyaline degeneration (Figures 6A, 6B).

**Figure 7:**
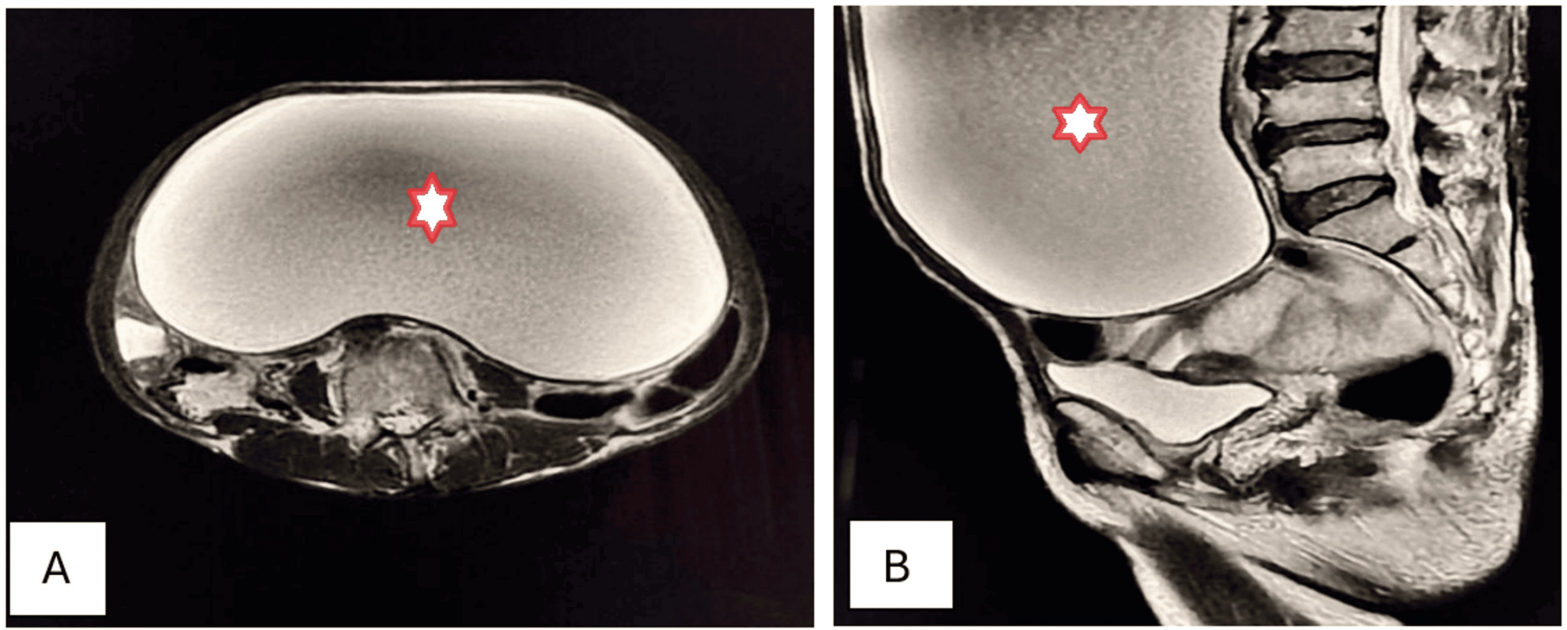
Serous cystadenoma of the right ovary (stars). Axial (A) and sagittal (B) T2W MRI shows a huge, well-circumscribed, unilocular, abdominopelvic, cystic mass arising from the pelvis and extending up to the upper abdomen with mass effect and peripheral displacement of the bowel loops, which is consistent with serous cystadenoma of the ovary. T2W: T2-weighted

A 53-year-old female presented to the OPD with complaints of distension and episodic pain in the right side of the abdomen, with bloating and constipation for the preceding five months. Physical examination revealed the presence of a soft, smooth, mobile, and large abdominopelvic mass. Ultrasonography identified a smooth-walled, unilocular, anechoic, huge cystic mass. Axial and sagittal T2W MRI confirmed the diagnosis of serous cystadenoma of the ovary (Figures 7A, 7B).

**Figure 8:**
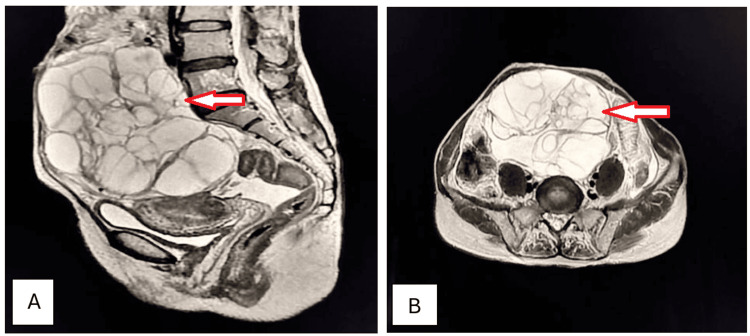
Mucinous cystadenoma of the right ovary (arrows). Sagittal (A) and axial (B) T2W MRI imaging shows a well-circumscribed, large, multiloculated, cystic mass in the pelvis with numerous internal septations, appearing predominantly hyperintense but of variable signal intensity because of the mucin content; the mass is overlying the uterus and causing a mass effect on the urinary bladder and uterus and displacing them inferiorly, which is consistent with a mucinous cystadenoma of the ovary. T2W: T2-weighted

A 45-year-old female presented to the OPD complaining of amenorrhea for the preceding five months with abdominal distension, chronic pelvic pain, increased urinary frequency, and constipation. Clinical examination disclosed a palpable abdominopelvic mass with dullness to percussion. Ultrasonography revealed a large, multilocular, cystic mass with thin septations and low internal echoes. Sagittal and axial T2W MRI detected multiloculated mucinous cystadenoma of the right ovary (Figures 8A, 8B).

**Figure 9:**
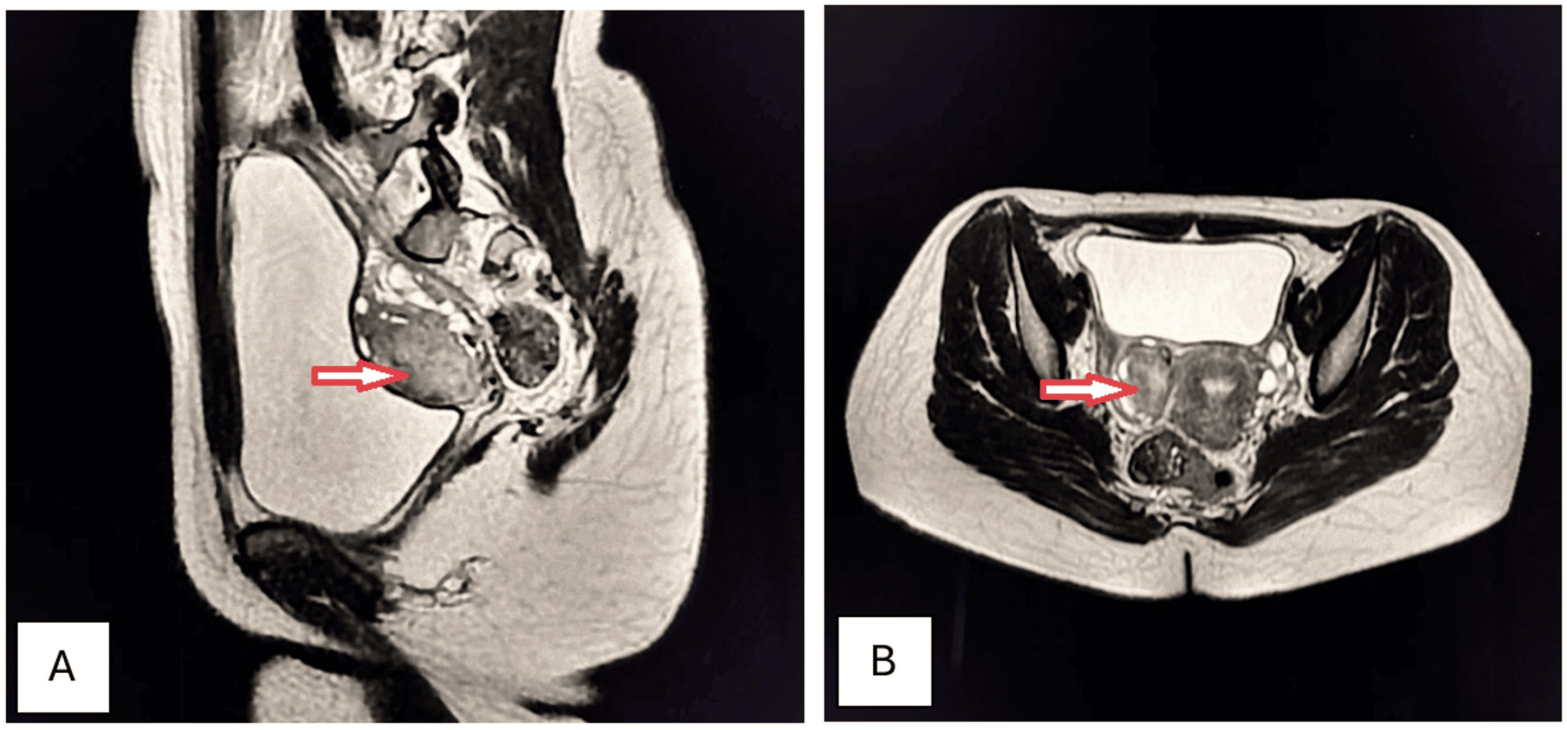
Fibroma of the right ovary (arrows). Sagittal (A) and axial (B) T2W MRI imaging shows a well-defined, round-to-oval, solid mass with a thick hypointense rim (black garland sign) with relative sparing of the central ovarian parenchyma. This iso- to hyperintense mass noted in the right ovary is displacing ovarian follicles peripherally, which is consistent with a fibroma. T2W: T2-weighted

A 42-year-old female came to the OPD with complaints of irregular menstruation and dull aching pelvic pain for six months. This was associated with increased urinary frequency. Ultrasonography identified the presence of a hypoechoic, solid, dark, adnexal mass with significant posterior acoustic shadowing. Sagittal and axial T2W MRI imaging identified the presence of fibroma of the right ovary (Figures 9A, 9B).

**Figure 10:**
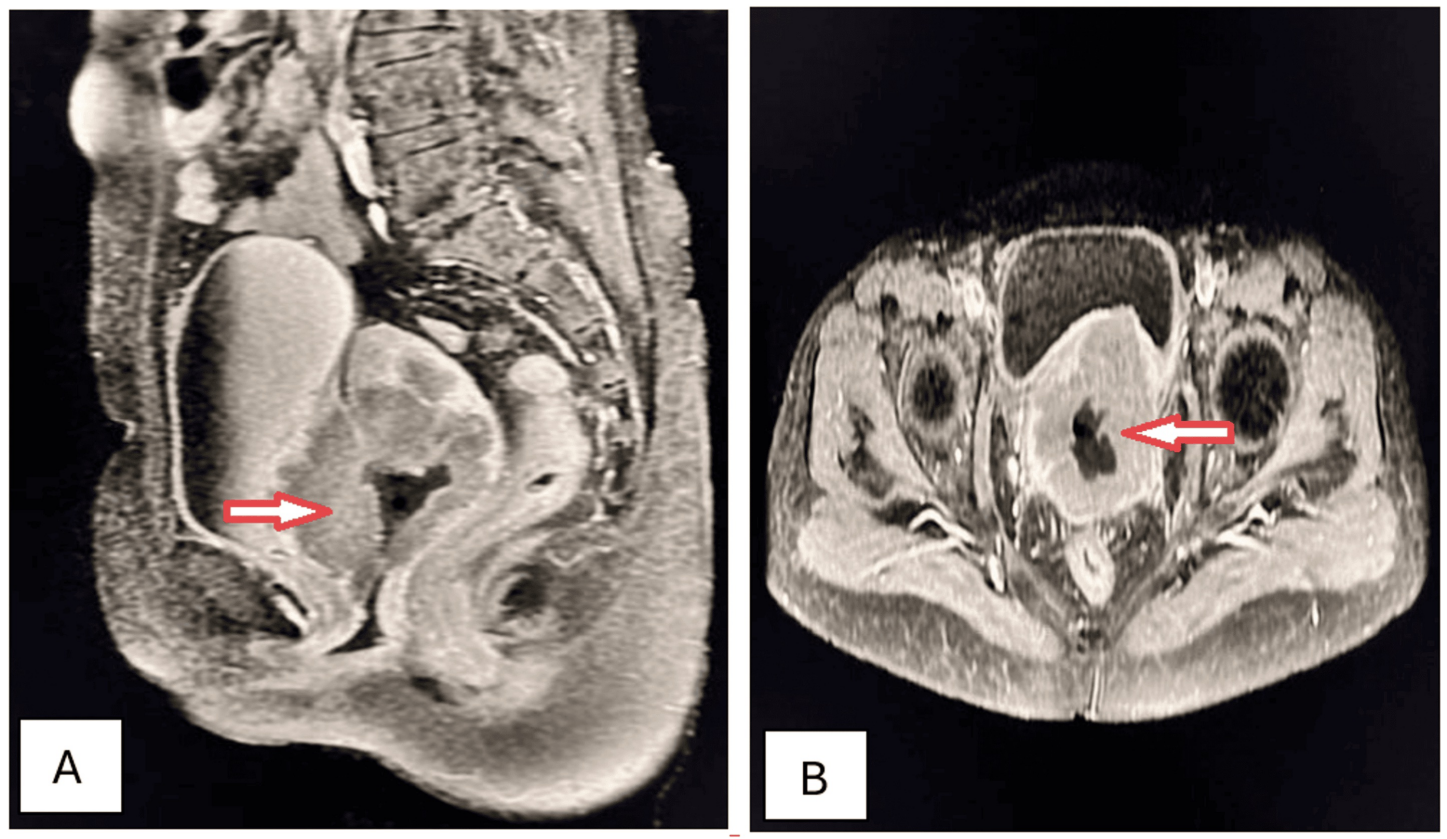
Infiltrating carcinoma of cervix (arrows). Sagittal (A) and axial (B) STIR imaging shows a well-circumscribed solid mass with lobulated margins appearing iso- to hyperintense with central hypointense necrosis in the anterior wall of the cervix and infiltration into the posterior bladder wall and posterior cervical wall, consistent with carcinoma cervix. STIR: short tau inversion recovery

A 43-year-old female presented to the OPD complaining of foul-smelling vaginal discharge and episodic, abnormal, post-coital vaginal bleeding for three months. It was associated with intermenstrual bleeding and persistent pain in the pelvic region. A Pap smear revealed a high-grade, squamous, intraepithelial lesion. Colposcopy disclosed thick, dense, chalky-white acetowhite lesions. Ultrasonography revealed a hypoechoic, heterogeneous mass involving the cervix. Sagittal and axial short tau inversion recovery (STIR) imaging detected a well-circumscribed solid mass as carcinoma cervix (Figures 10A, 10B).

**Figure 11:**
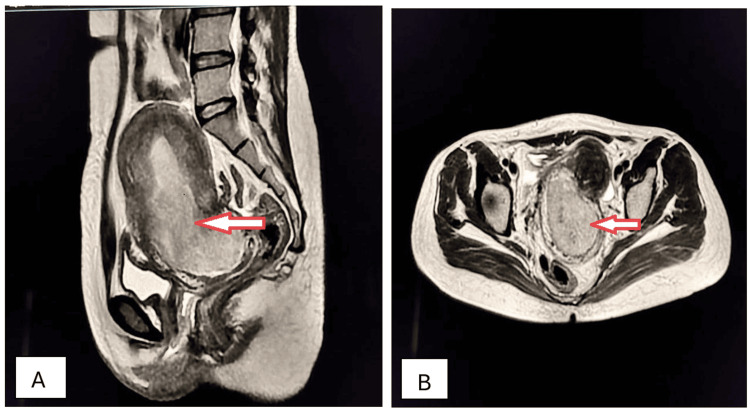
Carcinoma of endometrium with anterior and posterior wall invasion (arrows). Sagittal (A) and axial (B) T2W MRI imaging shows a well-circumscribed, inhomogeneous, predominantly hyperintense mass in the endometrial cavity and extending into the lower uterine segment and cervical canal, causing thinning of the cervical walls with anterior and posterior wall infiltration, consistent with carcinoma endometrium. T2W: T2-weighted

A 56-year-old postmenopausal female visited the OPD with complaints of abnormal vaginal bleeding and foul-smelling, bloody vaginal discharge for six months. These symptoms were associated with episodic cramping pain in the lower abdomen and increased frequency of urination. Transvaginal ultrasonography revealed a heterogeneous mass with mixed echo patterns and a thickened endometrial stripe exceeding 5 mm. Sagittal and axial T2W MRI imaging identified carcinoma endometrium, which was infiltrating the anterior and posterior walls of the uterus (Figures 11A, 11B).

**Figure 12:**
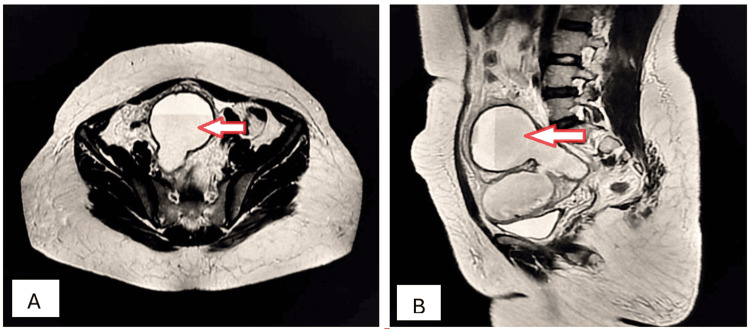
Endometrioma of right ovary (arrows). Axial (A) and sagittal (B) T2W MRI imaging shows a multiloculated, well-circumscribed, cystic mass in the pelvis and a shading sign with a fluid-hem level due to recurrent hemorrhage, consistent with endometrioma. T2W: T2-weighted

A 34-year-old female presented with complaints of persistent chronic pelvic pain with intense menstrual cramps and squeezing pain during sexual intercourse for five months. Transvaginal ultrasonography found a multilocular cystic lesion with ground glass echogenicity (reflecting old blood or chocolate-like fluid). Axial and sagittal T2W MRI imaging detected the presence of endometrioma (Figures 12A, 12B).

**Figure 13:**
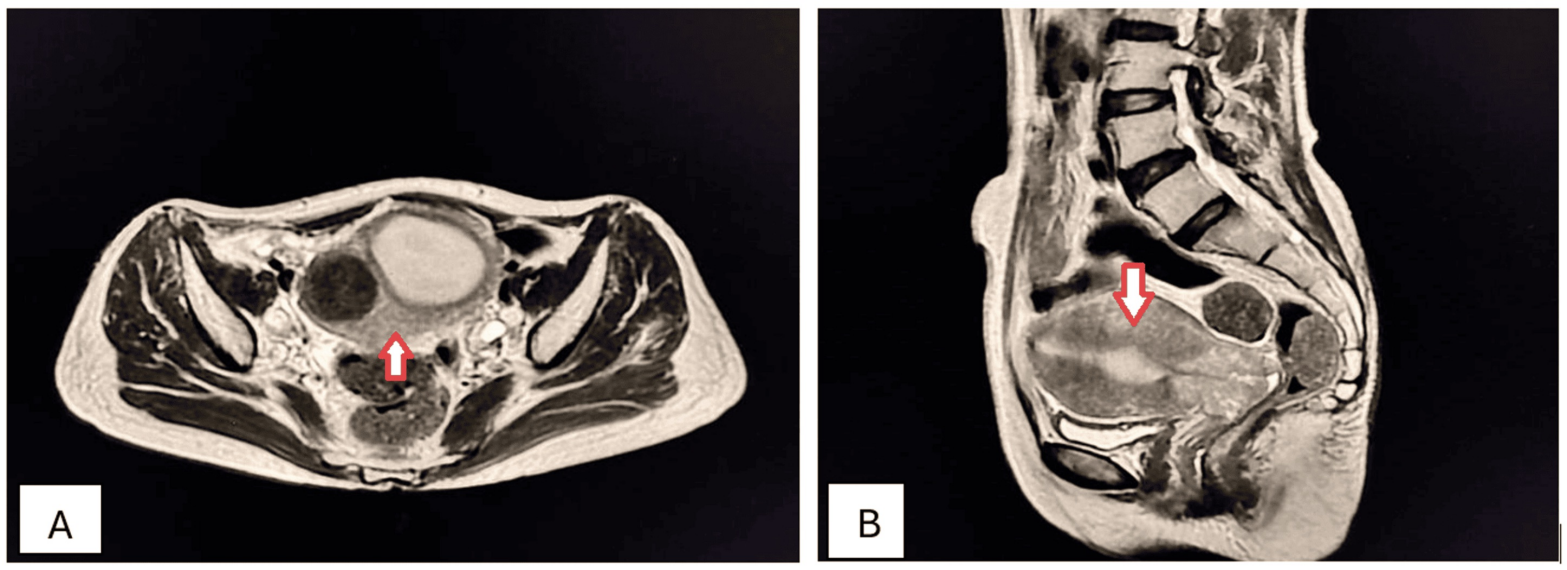
Adenomyosis of uterus (arrows) with cervical stenosis. Axial (A) and sagittal (B) T2W MRI shows an enlarged uterus with disproportionate enlargement of the posterior myometrium compared to the anterior myometrium, as well as numerous tiny cystic spaces in the transitional zone, consistent with adenomyosis. Similar changes are also observed in the cervix, consistent with cervical adenosis. A small, round, hypointense mass noted in the uterine myometrium on the right side is consistent with a fibroid. Adjacent to it on the left side, there is a well-circumscribed iso- to hyperintense endometrial collection due to cervical stenosis. T2W: T2-weighted

A 38-year-old female presented to the OPD with complaints of heavy, prolonged, painful menstrual bleeding for seven months. In between menstrual periods, she suffered persistent chronic pelvic pain. Physical examination, during palpation, disclosed an enlarged, boggy uterus that was tender to the touch. There was normocytic, normochromic anemia in the blood investigations. Ultrasonography revealed a globular, enlarged uterus with a heterogeneous myometrium including myometrial cysts. Axial and sagittal T2W MRI identified adenomyosis with an enlarged uterus (Figures 13A, 13B).

**Figure 14:**
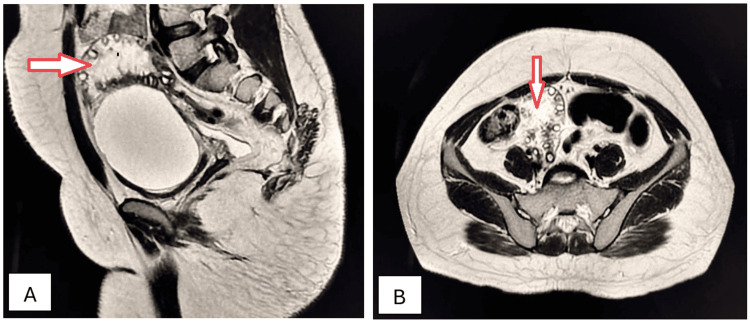
Enlarged right polycystic ovary (arrows). Sagittal (A) and axial (B) T2W MRI imaging shows an enlarged right ovary with small, sub-centimeter, peripheral follicles arranged in a necklace pattern with no dominant follicle and prominent, hyperintense central stroma, consistent with a polycystic ovary. T2W: T2-weighted

A 29-year-old obese female presented to the OPD complaining of infrequent menstruation (oligomenorrhea), excessive hair growth on the face and upper back, and weight gain during the preceding year. There were also dark patches on the neck and armpits. Ultrasonography revealed an enlarged right ovary (volume >10 mL) with small peripheral follicles creating the characteristic “string of pearls” appearance. Sagittal and axial T2W MRI imaging revealed the presence of polycystic ovaries (Figures 14A, 14B).

**Figure 15:**
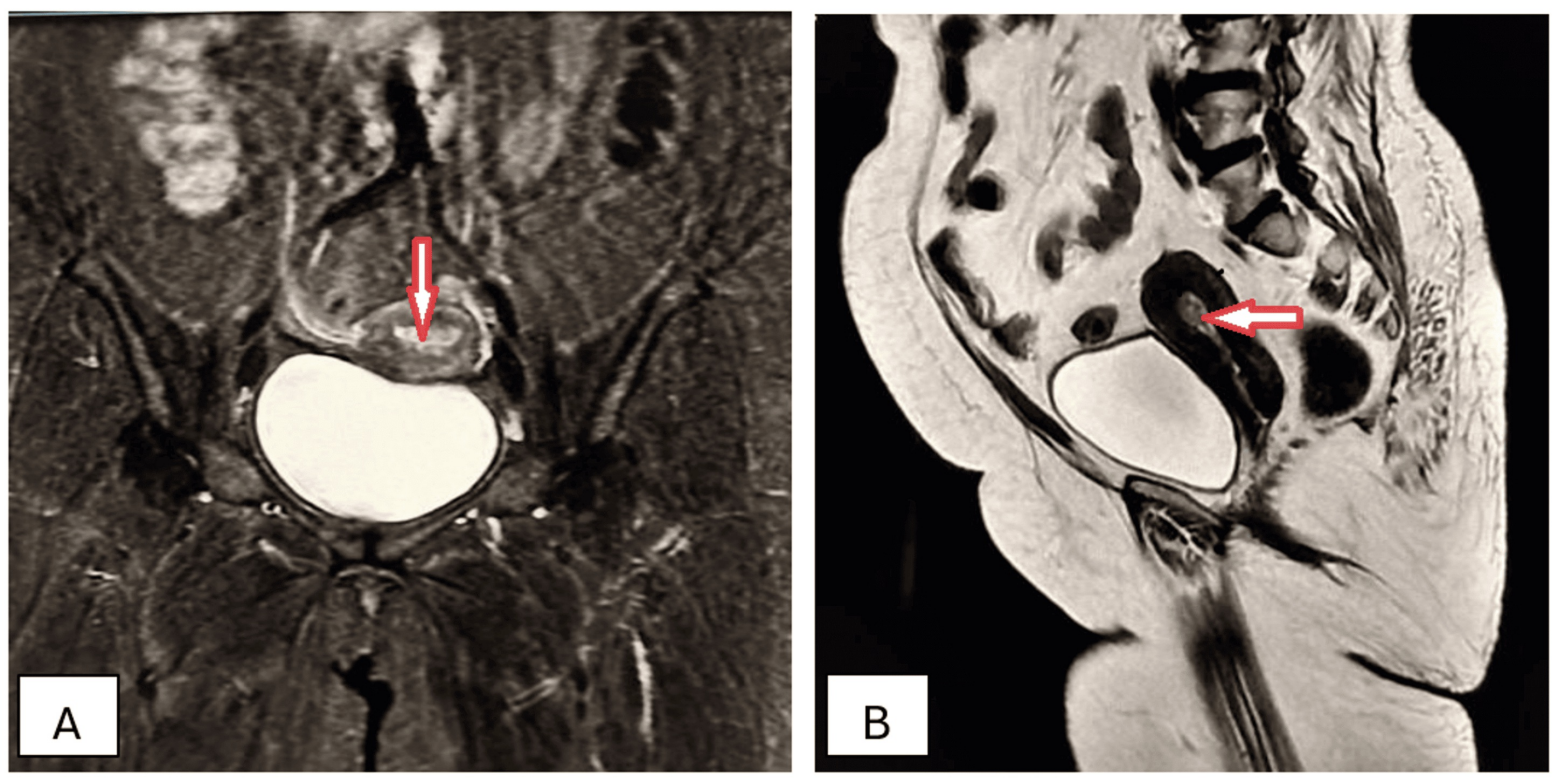
Sessile polyp of endometrium (arrows). Coronal STIR (A) and sagittal (B) T2W MRI imaging show a well-defined isointense mass in the endometrial cavity attached to the anterior wall, consistent with a sessile endometrial polyp. T2W: T2-weighted; STIR: short tau inversion recovery

A 40-year-old female came to the OPD with complaints of heavy menstrual bleeding for nine months, which was associated with spotting between periods. Ultrasonography showed a well-defined hyperechoic mass within the endometrial cavity. Additional Doppler revealed a single feeding vessel (pedicle artery sign). Coronal STIR and sagittal T2W MRI imaging identified the presence of an endometrial polyp (Figures 15A, 15B).

**Figure 16:**
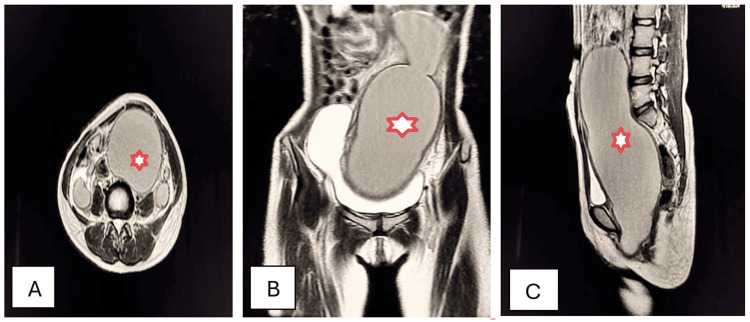
Hematometra and hematocolpos due to imperforate hymen (stars). Axial (A), coronal (B), and sagittal (C) T2W MRI imaging shows a huge enlargement of the uterus with thinning of the myometrial and cervical walls due to hypointense endometrial and endocervical collection (with the shading sign due to recurrent hemorrhage), consistent with hematometra and hematocolpos caused by an imperforate hymen. T2W: T2-weighted

A 14-year-old female presented to the OPD with complaints of cyclic pelvic pain and absence of a first menstrual period. Two episodes of acute urinary retention were medically managed. There was associated constipation with episodes of a strong urge to defecate. Physical examination revealed a palpable mass in the lower abdomen. During a vaginal examination, a bluish, swollen membrane was visible. Ultrasonography identified the presence of a large, fluid-filled distension of the vagina and uterus due to retained menstrual blood, appearing as a cystic hypoechoic mass. Axial, coronal, and sagittal T2W MRI detected the presence of an imperforate hymen with hematometra and hematocolpos (Figures 16A-16C).

**Figure 17:**
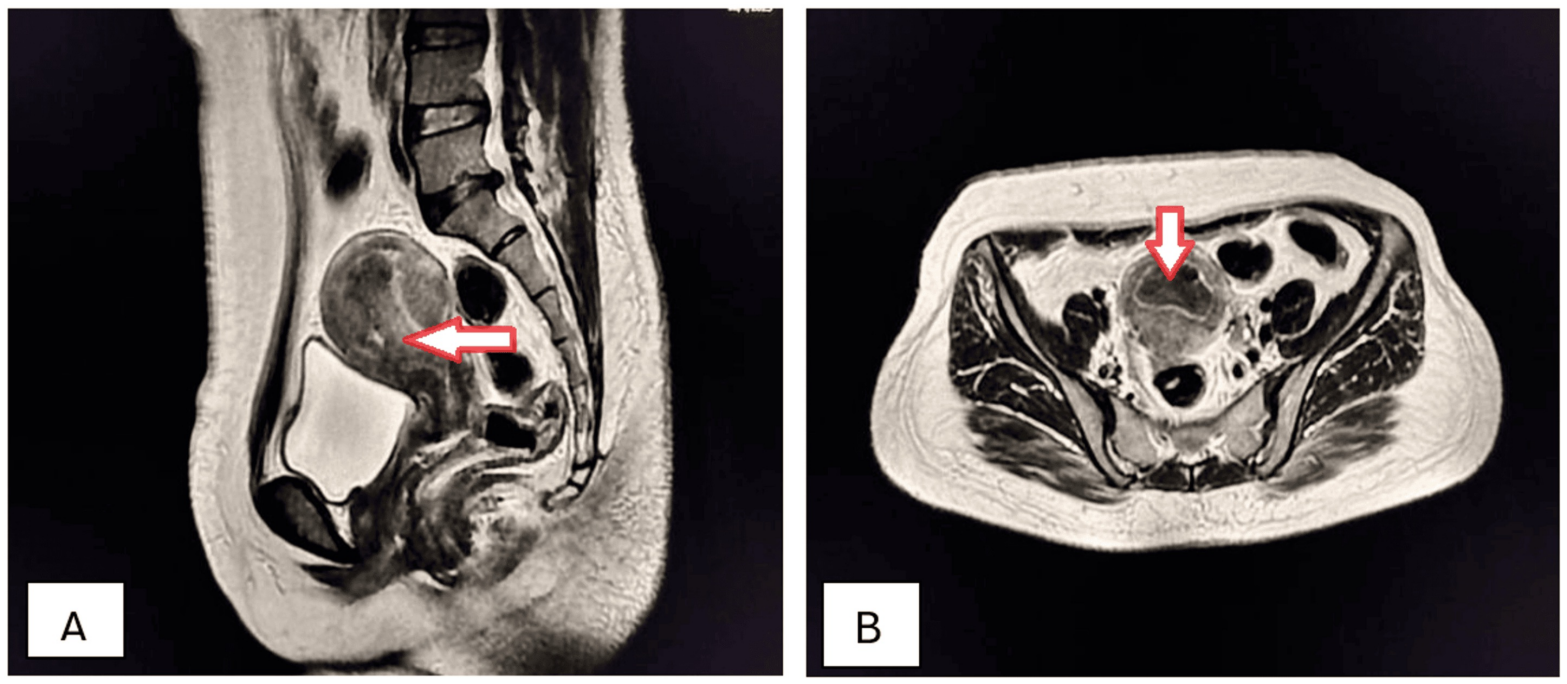
Retained products of conception (arrows). Sagittal (A) and axial (B) T2W MRI imaging shows inhomogeneous endometrial collection with peripheral, hyperintense endometrial lining, consistent with retained products of conception (an incomplete abortion). T2W: T2-weighted

A 31-year-old female came to the OPD with complaints of sudden, heavy vaginal bleeding with severe cramps and foul-smelling vaginal discharge. There was a history of miscarriage 10 days before these symptoms. Ultrasonography revealed a discrete, hyperechoic, heterogeneous mass within the endometrial cavity, accompanied by a thickened endometrium. Sagittal and axial T2W MRI imaging detected retained products of conception (Figures 17A, 17B).

## Discussion

Currently, MRI is the only reliable diagnostic approach for primary diagnosis and characterization of gynecological masses. This non-invasive modality assures no exposure to radiation, no need for an anesthetic agent, and minimal dependency on the operator. MRI not only identifies the anatomical origin and shape of the lesion but simultaneously provides excellent information about tissue features like the presence of fluid, blood, fat, fibrosis, smooth muscle, and lymphoid and myxoid tissues.

The sample size of 106 cases of gynecological masses that were included in this retrospective study was optimum compared to other relevant studies [12,13]. In our study, the gynecological masses were found in females from their second to eighth decades of life. The highest incidence of gynecological masses occurred in the fourth and fifth decades. This is comparable to a study performed by Moideen et al. [14]. The incidence of benign neoplasms and non-neoplastic masses was in ascending order up to the fifth decade, after which there was a decline in the incidence of both masses. In contrast, most malignant masses were found in the sixth decade. Similar findings were observed in a study performed by Mathew et al. [15].

Clinically, the most common findings for the benign neoplasms were pain in the abdomen, menstrual irregularities, and abdominal distention. In contrast, postmenopausal bleeding and vaginal discharge were common in malignant neoplasms. Non-neoplastic masses had variable presentations, with three masses diagnosed incidentally.

Uterine leiomyomas were the most common gynecological mass in our study, and similar findings were observed in a previous study [16]. MRI clearly calculated the number, size, and anatomical origin of fibroids in this study. Most of the lesions were well-defined, round-to-oval, hypointense masses with some having a mass effect on the adjoining bladder or endometrial canal. MRI was accurate in identifying hyaline, cystic, fatty, and hemorrhagic changes in the fibroids. Calcification was identified by low signal intensity, and vascularity was identified in some fibroids on T2W imaging by identifying flow voids. This could help surgeons plan embolization prior to myomectomy. The same was observed in a study conducted by Schwartz et al. [17].

The next most common benign neoplasms were epithelial ovarian tumors and fibromas of the ovary. MRI precisely differentiated serous, mucinous, solid, cystic, fatty, hemorrhagic, and fibrous components of ovarian masses. In this study, MRI identified serous cystadenomas as large, well-circumscribed, unilocular or multilocular, abdominopelvic cystic masses with homogeneous fluid within and high signal intensity on T2W imaging. In some cases, these lesions had a mass effect on the bowel loops and urinary bladder. In contrast, mucinous cystadenomas appeared as well-circumscribed, large, multiloculated cystic masses in the pelvis with numerous internal septations, predominantly hyperintense on T2-weighted images but with variable signal intensity due to mucin content; most had mass effect on the uterus and urinary bladder. Lack of fat suppression distinguishes these lesions from dermoid cysts, whereas the lack of solid elements differentiates these from adenocarcinomas. The same findings were observed by Seo et al. [18]. The diagnosis of ovarian fibroma was confirmed by the presence of well-circumscribed, round-to-oval, solid masses with low signal intensity on T2W images due to the presence of dense fibrous tissue. A thick hypointense rim (black garland sign) with relative sparing of the central ovarian parenchyma was present. This iso- to hyperintense mass was displacing ovarian follicles peripherally. These findings are similar to the results of a previous study [19].

Among the malignant neoplasms, cervical carcinoma was diagnosed in nine patients either in their fifth or sixth decade of life. In a study conducted by Mahajan et al., most occurrences of cervical carcinomas were noticed in females in their fourth decade [20], though in a study performed by Shweel et al., the results were comparable to this study, with a peak incidence of cervical cancer in the fifth decade of life [21]. In the present study, MRI provided information about size, shape, volume, local spread, such as in stromal and parametrial invasion, and lymph node status, and it helped in planning the next mode of treatment. Both the exophytic and endocervical masses were clearly identified by T2W images with intermediate-to-high signals. A study conducted by Kim et al. made similar observations [22].

The highest incidence of endometrial carcinoma was found in elderly females in their sixth and seventh decades of life, consistent with the study by Arora and Quinn [23]. Most of the lesions appeared as irregular or polypoid endometrial masses with mild hyperintensity compared to a normal uterus. MRI helped us identify the presence of myometrial invasion, cervical extension, and vaginal and lymph node involvement precisely, consistent with the study by Xu-Welliver et al. [24]. In this study, the single occurrence of a mucinous cystadenocarcinoma of the ovary in a patient in her eighth decade was easily identified by the presence of necrosis, thickened wall, peritoneal deposits, and pelvic wall extension, consistent with the study by Antonio et al. [25].

Among the non-neoplastic masses, endometriosis was the most common, accounting for 19 cases. In the present study, the endometriomas were completely different from other lesions because of the presence of different stages of hemorrhage and protein particles, which were bright in T2W images with higher intensities. In the same images, fibrotic lesions appeared as dark, spiculated masses with irregular borders, indicating adhesions. Similar findings were observed in a study performed by Outwater et al. [26]. This grouping was followed by nine cases of adenomyosis. MRI findings revealed a thickened junctional zone of more than 12 mm along with the characteristic hyperintense bright spots on T2W images. These indicated the presence of cysts with hemorrhage. T1 images identified the presence of ectopic glandular tissue within the myometrium. In addition, the T2W images revealed the presence of an enlarged and globular uterus with adenomyomas. These features differentiated them from fibroids. Comparable findings were confirmed in a study conducted by Taran et al. [27].

Among the ovarian cysts, T2W MRI revealed an enlarged ovary with small, sub-centimeter, peripheral follicles arranged in a necklace pattern with no dominant follicle and prominent hyperintense central stroma. In one case, there was a distinct dark rim around the ovary, which indicated fibrosis of the capsule. A previous study depicted the same features [18]. Endometrial polyps also showed distinct features. These typically looked like focal sessile masses in the uterine cavity with T2 hyperintensity in the periphery and central, hypointense, dark, fibrous core, with cysts inside the masses representing vascular and fibrous tissues. The findings are consistent with a previous study [28]. There were two cases of hematocolpos due to the presence of an imperforate hymen. MRI revealed a huge enlargement of the uterus with thinning of myometrial and cervical walls caused by hypointense, endometrial and endocervical collection (shading sign due to recurrent hemorrhage), which is consistent with hematometra and hematocolpos. Previous research established similar findings [29]. The final category of non-neoplastic masses was a case of retained products of conception. This appeared as an inhomogeneous, irregular mass within the endometrial cavity with heterogeneous signal intensity on the T2W imaging, which indicated the presence of vascularized, retained placental tissues consistent with the retained products of conception. A study conducted by Noonan et al. described similar features [30].

In the present study, though MRI provided detailed information about various uterine and adnexal masses, there are still some shortcomings. The findings could not be correlated or established through histopathological examination because of ethical issues related to social beliefs of patients and their families, fear of invasive procedures, the financial backgrounds of the patients, and laboratory-related issues. The current study was conducted at a single tertiary care center and had a limited sample size. Therefore, we cannot apply the incidences and characteristics of the gynecological masses to the whole population. The study was observational and retrospective in nature. If it had been a prospective study, follow-up would also have been possible. A revised research protocol, prospective in nature with a larger sample size involving multiple tertiary care centers, could explore MRI findings in different populations with different age groups and clinical presentations. This would surely prove the role of MRI in diagnosing and characterizing gynecological masses.

## Conclusions

In the present study, MRI was found to be an excellent tool for identifying uterine and adnexal masses. MRI clearly differentiated benign, malignant, and non-neoplastic masses in different age groups. It provided precise information about the origin, size, shape, and consistency of gynecological masses. Moreover, MRI provided information about the mass effects of lesions and their relation to surrounding structures. In malignant masses, MRI correctly provided the extent of the local invasion and the status of the lymph nodes. Hence, planning the next line of management and selecting the best treatment option was feasible. Even after treatment, further timely follow-up of pelvic anatomy was attainable because this diagnostic modality lacks the risk of radiation.
